# Complete Genome Sequences and Methylome Analyses of Cutibacterium acnes subsp. *acnes* Strains DSM 16379 and DSM 1897^T^

**DOI:** 10.1128/MRA.00705-20

**Published:** 2020-07-16

**Authors:** Paulina Deptula, Pia Laine, Lars Paulin, Petri Auvinen, Richard J. Roberts, Christopher D. Johnston, Pekka Varmanen

**Affiliations:** a Department of Food and Nutrition, University of Helsinki, Helsinki, Finland; b Institute of Biotechnology, University of Helsinki, Helsinki, Finland; c New England BioLabs, Ipswich, Massachusetts, USA; d Fred Hutchinson Cancer Research Center, Seattle, Washington, USA; University of Rochester School of Medicine and Dentistry

## Abstract

Cutibacterium acnes is a member of the normal human skin microbiome. However, it is also associated with skin disorders and persistent infections of orthopedic implants. Here, we announce complete genome sequences and methylomes of the *C. acnes* subsp. *acnes* strains DSM 1897^T^ and DSM 16379 together with their active restriction-modification systems.

## ANNOUNCEMENT

Cutibacterium acnes was recently reclassified from Propionibacterium acnes, when the former genus *Propionibacterium* was divided into four new genera, namely, *Propionibacterium*, *Cutibacterium*, *Acidipropionibacterium*, and *Pseudopropionibacterium* (1). The strain *C. acnes* subsp. *acnes* DSM 1897^T^ was isolated from acne pustules on facial skin in 1920 (2). The strain *C. acnes* subsp. *acnes* DSM 16379, which is equivalent to the first sequenced representative of the former genus *Propionibacterium* (3) strain KPA 171202, was isolated as a contaminant of an anaerobic culture (4). Both strains belong to the former type I, with the strain DSM 16379 representing subtype IB, while the strain DSM 1897^T^ belongs to subtype IA_1_ (5).

The strains were anaerobically grown in brain heart infusion broth at 37°C. Genomic DNA from stationary-phase cultures was isolated with the DNA minikit (Qiagen, Germantown, MD, USA). SMRTbell DNA libraries were prepared without shearing, using the PacBio DNA/polymerase binding kit P5 and DNA template preparation kit 3.0 (Pacific Biosciences, USA) according to the manufacturer’s protocol. A sample from DSM 1897^T^ was size selected (4 kb) using the BluePippin system (Sage Sciences). Libraries were sequenced with P5/C3 chemistry on a PacBio RS II sequencer (Pacific Biosciences), resulting in 140,910 (*N*_50_, 14,274 bp) and 105,136 (*N*_50_, 20,157 bp) polymerase reads for DSM 16379 and DSM 1897^T^, respectively. Genome assemblies (HGAP3) and motif and modification analyses implemented in SMRT Portal 2.3.0 (Pacific Biosciences) were performed using default parameters. Genome sequences were circularized using GAP4 (Staden package) (6), resulting in genomes with sizes of 2,495,002 bp (G+C content, 60.0%; coverage, 446×; GenBank accession number CP025934) and 2,560,634 bp (G+C content, 60.0%; coverage, 351×; GenBank accession number CP025935) for DSM 1897^T^ and DSM 16379, respectively. One methylation motif was detected for each of the strains. Annotation with the NCBI Prokaryotic Genome Annotation Pipeline (PGAP) (7) and SEQWARE methylome analysis at REBASE (8) were performed as previously described (9).

**FIG 1:**
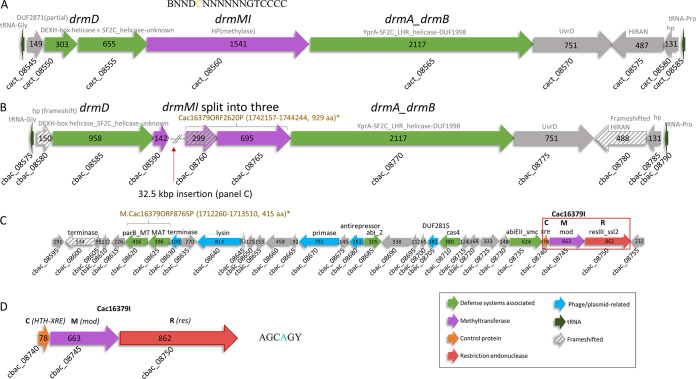
Restriction-modification (RM) systems identified in *C. acnes* subsp. *acnes* strains. (A) DISARM in strain DSM 1897^T^. An 89% identical gene locus is present in a member of the third subspecies of *C. acnes*, namely, *C. acnes* subsp. *elongatum* JCM 18919^T^ (GenBank accession number BFFM01000008.1; nucleotides 185832 to 205117) (formerly *C. acnes* type III) (5, 13). (B) Remnant of the DISARM in strain DSM 16379. The 32.5-kbp insertion is indicated. The DISARM genes and 32.5-kbp insertion are absent from *C. acnes* subsp. *defendens* ATCC 11828^T^ (formerly *C. acnes* type II). (C) Genomic island disrupting the DISARM in DSM 16379 containing 33 putative open reading frames (ORFs) (cbac_08595 to cbac_08755) and which splits the *drmMI* gene into three parts (cbac_08590, cbac_08760, and cbac_08765). The active type III RM system is indicated. (D) Type III RM system in strain DSM 16379. The recognition motif assigned to the Mod-type methylase of the system is AGCAGY, and 100% of the motifs in the genome were methylated. *, gene coordinates and product sizes of the putative methylases predicted at REBASE.

The methylation motif BNNDCNNNNNNGTCCCC detected in the strain DSM 1897^T^ cannot be genuine and may reflect the presence of an unusual modification for which the PacBio motif detection software was not trained, which could explain the low detection rate of 24.1%. One possibility is that it reflects a further modification of a methylation that is associated with a system strongly resembling a defense island system associated with restriction-modification (DISARM) (10) (Fig. 1A). If the type IIG system Cac1897ORF8560P is responsible, then one of the neighboring genes may provide the enzymatic activity to further modify the true motif. Further studies are needed to determine the true character of this possible DISARM.

A similar system resembling the DISARM is present in the strain DSM 16379 (Fig. 1B) but is disrupted by a 32.5-kbp insertion (Fig. 1C). The 32.5-kbp insertion carries multiple genes with putative roles in defense, a typical characteristic of a “defense island” (11), and it includes an active type III restriction-modification (RM) system (cbac_08740 to cbac_08750) (Fig. 1D) with a recognition motif, AGCAGY. A similar type III system with the same recognition motif was previously reported in a closely related bacterium, Propionibacterium freudenreichii (12).

### Data availability.

The genome sequences are available at NCBI with accession numbers CP025934.1 and CP025935.1, and the methylome analyses are available at REBASE with organism numbers 23064 and 23063 for strains DSM 1897^T^ and DSM 16379, respectively. The BioProject accession numbers are PRJNA429715 and PRJNA429713.
